# Excess generation and activation of naturally arising memory-phenotype CD4^+^ T lymphocytes are inhibited by regulatory T cells in steady state

**DOI:** 10.3389/fimmu.2024.1429954

**Published:** 2024-08-16

**Authors:** Jing Li, Ziying Yang, Akihisa Kawajiri, Kosuke Sato, Shunichi Tayama, Naoto Ishii, Jinfang Zhu, Takeshi Kawabe

**Affiliations:** ^1^ Department of Microbiology and Immunology, Tohoku University Graduate School of Medicine, Sendai, Miyagi, Japan; ^2^ Molecular and Cellular Immunoregulation Section, Laboratory of Immune System Biology, National Institute of Allergy and Infectious Diseases, National Institutes of Health, Bethesda, MD, United States

**Keywords:** CD4 T lymphocytes, memory, homeostasis, regulatory T cells, inflammation

## Abstract

Conventional CD4^+^ T lymphocytes consist of naïve, foreign antigen-specific memory, and self-antigen-driven memory-phenotype (MP) cell compartments at homeostasis. We recently showed that MP cells tonically proliferate in response to self-antigens and differentiate into the T-bet^+^ subset in steady state. How excess proliferation and differentiation of MP cells are inhibited remains unclear. Given immunosuppressive function of regulatory T cells (Tregs), it is possible that they are also involved in inhibition of spontaneous MP cell activation. Here we show using Foxp3-diphtheria toxin receptor-transgenic mice that both MP and naïve CD4^+^ T cells spontaneously proliferate and differentiate into Th1 cells upon acute Treg depletion. At an early time point post Treg depletion, MP as compared to naïve CD4^+^ T cells are preferentially activated while at a later stage, the response is dominated by activated cells originated from the naïve pool. Moreover, we argue that MP cell proliferation is driven by TCR and CD28 signaling whereas Th1 differentiation mediated by IL-2. Furthermore, our data indicate that such activation of MP and naïve CD4^+^ T lymphocytes contribute to development of multi-organ inflammation at early and later time points, respectively, after Treg ablation. Together our findings reveal that Tregs tonically inhibit early, spontaneous proliferation and Th1 differentiation of MP CD4^+^ T lymphocytes as well as late activation of naïve cells, thereby contributing to maintenance of T cell homeostasis.

## Introduction

Conventional CD4^+^ T lymphocytes are essential for adaptive immune responses and composed of naïve (CD44^lo^ CD62L^hi^), foreign antigen (Ag)-specific memory, and self Ag-driven “memory-phenotype (MP)” cells (both CD44^hi^ CD62L^lo^) in steady state (1, 2). In pathogen infection, naive cells that have T cell receptors (TCRs) specific for cognate foreign Ags are activated to differentiate into effector cells to contribute to host protection. After the pathogen concerned is eliminated most effector cells die, leaving a population of foreign Ag-specific memory cells that provides the host with immunological memory.

In addition to the above conventional T cell activation pathway, naïve cells can weakly respond to self Ags to spontaneously acquire a memory phenotype in steady state. Thus, in unimmunized mice housed in a specific pathogen-free (SPF) environment, approximately 10% of Foxp3^−^ CD4^+^ T lymphocytes adopt a CD44^hi^ CD62L^lo^ phenotype (3). Because these cells are equally present in SPF, germ-free (GF), and antigen-free (AF) animals that virtually lack all foreign Ags derived from commensal microbiota and food (3, 4), the majority of the CD44^hi^ CD62L^lo^ CD4^+^ T cell population represents self Ag-driven MP rather than foreign Ag-specific memory cells (5).

Because of their self-reactivity, MP cells tonically proliferate through “homeostatic proliferation” in the periphery; indeed, ~30% of MP cells are in cell cycle and thus Ki67^+^ at homeostasis (6, 7). In addition, we previously reported that MP cells homeostatically differentiate into the “Th1-like” T-bet^+^ subset in steady state and that this subpopulation can exert innate effector function in pathogen infection by producing IFN-γ in response to IL-12 in the absence of foreign Ag recognition (3, 8). These observations suggest that MP cells may have a potential to induce inflammatory responses in certain circumstances. Because the same cells do not induce immunopathology in healthy conditions, such inflammatogenic nature of MP cells is thought to be inhibited by some mechanisms. However, it remains to be determined how excessive proliferation and Th1 differentiation of MP cells are regulated in steady state.

Foxp3^+^ regulatory T cells (Tregs) play an important role in inhibition of several immune responses (9). Indeed, it has been reported that acute Treg removal results in unregulated CD4^+^ T cell activation and following inflammation and that this T cell overactivation can be triggered in the absence of foreign Ags (10–12), demonstrating that Tregs tonically suppress T cell responses to available Ags including self in steady state. Mechanistically, in conventional, foreign Ag-directed T cell immune responses, Tregs can inhibit Ag-specific CD4^+^ T cell activation by depleting peptide-major histocompatibility complex class II (MHC II) complexes from antigen-presenting cells (APCs) including dendritic cells (DCs) (13). In addition, Tregs can exert their suppressive function by capturing CD80 and CD86 on APCs (14). Furthermore, Tregs can constraint T cell activation by consuming IL-2 produced by activated CD4^+^ T cells (15, 16). However, it is unknown whether Tregs can differentially suppress MP vs. naïve CD4^+^ T lymphocytes in steady state, and if so, what mechanisms govern such Treg responses to these two types of T cells.

In the present study, we have investigated whether homeostatic proliferation and differentiation of MP vs. naïve CD4^+^ T cells are inhibited by Tregs in steady state and whether molecular mechanisms dictating Treg-mediated inhibition of T cell proliferation vs. differentiation are similar or different. Our results show that Tregs tonically suppress overactivation of MP cells by restricting their proliferation and differentiation. While Tregs also suppress the activation of naïve cells, the kinetics are different. Furthermore, we argue that Treg-mediated inhibition of MP cell proliferation depends on TCR and CD28 signals whereas that of Th1 differentiation is mediated through IL-2 signaling. Together our results reveal essential roles for Tregs in inhibition of inflammatogenic potential of MP CD4^+^ T lymphocytes and thus in maintenance of their homeostasis.

## Results

### MP vs. naive CD4^+^ T lymphocytes more efficiently generate new MP cells at an early time point when Tregs are acutely depleted

**Figure 1:**
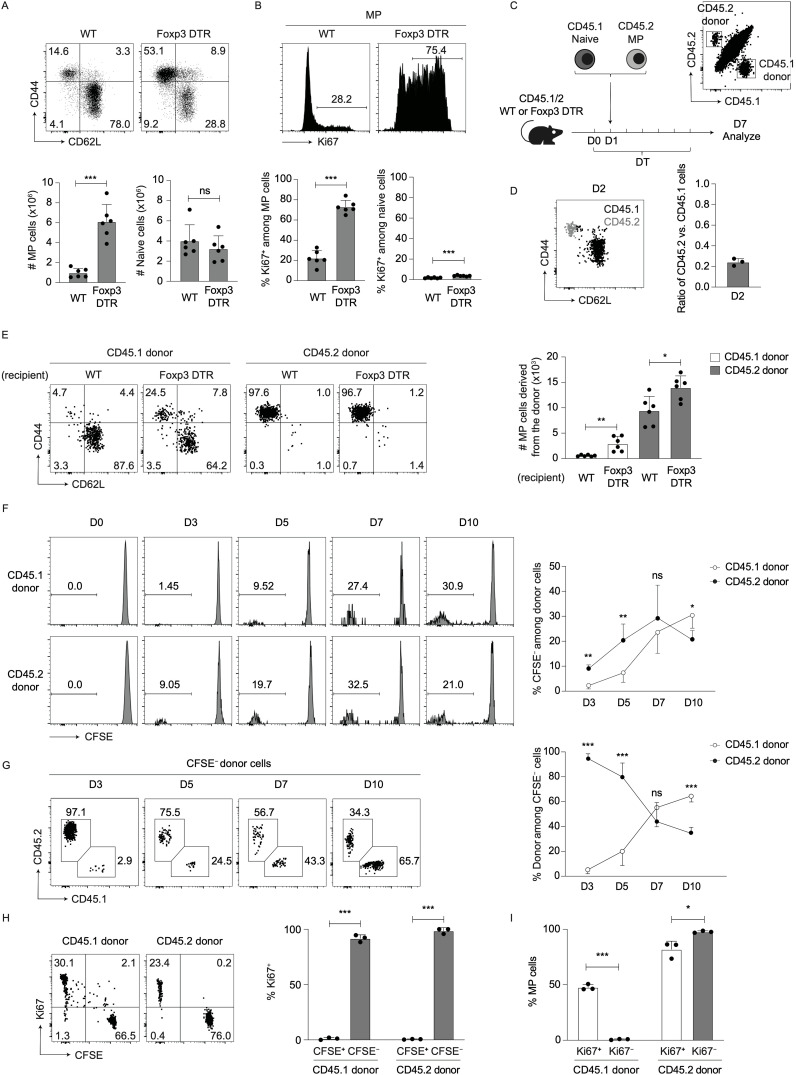
Both preexisting MP and naïve CD4^+^ T lymphocytes rapidly proliferate to give rise to new MP cells in the environment where Tregs are acutely depleted. **(A, B)** The MP cell population expands in size after Treg depletion. Foxp3-DTR and WT mice were injected with DT for 7 consecutive days and splenocytes analyzed on day 7. **(A)** The representative dot plots indicate expression of CD44 and CD62L in Foxp3^−^ CD4^+^ T cells while the bar graphs show the number (mean ± SD) of MP and naïve CD4^+^ T cells from each group (n=6). **(B)** The representative histograms display Ki67 expression in MP CD4^+^ T lymphocytes whereas the bar graphs indicate the frequency (mean ± SD) of Ki67^+^ cells among MP and naïve cells (n=6). Data shown are pooled from two independent experiments performed. **(C–E)** Both MP and naïve CD4^+^ T cells can contribute to new MP cell generation when Tregs are ablated. **(C)** Experimental design. MP and naïve CD4^+^ T cells were sorted out from CD45.2 and CD45.1 mice, respectively, mixed at a 1:1 ratio, and transferred into CD45.1/2 Foxp3-DTR or WT mice that were subsequently subjected to DT treatment. Splenic donor cells were analyzed at different time points. **(D)** The overlaid dot plot shows CD44 and CD62L expression in CD45.1 and CD45.2 donor cells (black: CD45.1 donor, gray: CD45.2 donor) whereas the bar graph indicates the ratio of CD45.2 vs. CD45.1 donor cells on day 2 (n=3). Data are representative of two independent experiments. **(E)** The representative dot plots display expression of CD44 and CD62L among donor cells while the bar graph shows the absolute number (mean ± SD) of MP cells generated from CD45.1 and CD45.2 donor populations (n=6). Data are pooled from two independent experiments. **(F–I)** Fast proliferation of MP and naïve CD4^+^ T lymphocytes generates new MP cells in the absence of Tregs. In the above experiments donor cells were measured for CFSE dilution as well as Ki67 expression. **(F)** The histograms show CFSE dilution of CD45.1 and CD45.2 donor cells at different time points while the graph indicates the frequency (mean ± SD) of CFSE^−^ cells among total donor cell population from each group (n=3-5). **(G)** Representative dot plots displaying CD45.1 and CD45.2 expression in CFSE^-^ total donor cells at the indicated time points together with a line graph showing the frequency (mean ± SD) of CD45.1 and CD45.2 donor cells among CFSE^-^ total donor population (n=3-5). Data are pooled from two independent experiments. **(H)** Representative dot plots displaying Ki67 expression and CFSE dilution in CD45.1 and CD45.2 donor cells on day 7 together with a bar graph showing the frequency (mean ± SD) of Ki67^+^ fraction among CFSE^+^ and CFSE^−^ donor populations (n=3). **(I)** A bar graph indicating the frequency (mean ± SD) of MP cells among Ki67^+^ and Ki67^−^ donor populations from each group (n=3). Data are representative of two independent experiments. * *p*<0.05, ** *p*<0.01, *** *p*<0.001, ns: not significant.

To address whether Tregs can inhibit MP cell proliferation in steady state, we used Foxp3-diphtheria toxin receptor-transgenic (Foxp3-DTR) mice where Foxp3^+^ Tregs can be transiently depleted by diphtheria toxin (DT) treatment (17). We first treated Foxp3-DTR mice with DT every day and analyzed Foxp3^+^ cells at different time points. As reported previously (17), DT treatment transiently and significantly reduced Tregs, followed by their recovery at later time points (**Supplementary Figure 1**). We next examined MP and naïve cells on day 7. As shown in **Figure 1A**, splenic MP but not naïve Foxp3^-^ CD4^+^ T cells increased in number by Treg depletion. The majority of MP cells acquired a Ki67^+^ phenotype whereas naïve cells remained Ki67^−^ (**Figure 1B**), confirming that the CD44^hi^ CD62L^lo^ Foxp3^-^ CD4^+^ T cell fraction expands in the environment where Tregs are acutely depleted.

Because naïve Foxp3^-^ CD4^+^ T lymphocytes can convert to MP cells and because MP cells themselves can further proliferate to maintain their homeostasis in the periphery (3), both preexisting naïve and MP cells could contribute to the above expansion of the CD44^hi^ CD62L^lo^ Foxp3^-^ CD4^+^ T cell population. To ask which population(s), preexisting naïve or MP cells, contribute to the expansion of CD44^hi^ CD62L^lo^ Foxp3^−^ CD4^+^ T cells, we sorted for naïve and MP cells from congenic CD45.1 and CD45.2 mice, respectively, mixed at a 1:1 ratio, and adoptively transferred these cells into CD45.1/2 hosts that were either WT or Foxp3-DTR (**Figure 1C**). These mice were subsequently subjected to DT treatment to deplete host-derived Tregs. While the same amounts of CD45.1 and CD45.2 donor cells were mixed at the time of transfer, the ratio of CD45.2 vs. CD45.1 donor cells that dwelled in the spleen of WT hosts 24 hours later was 1:5 (**Figure 1D**). Given that a majority of donor cells accumulated in the spleen but not in extra-lymphoid organs (**Supplementary Figure 2**), this dwelling ratio presumably reflects the lower capacity of MP as compared to naïve cells to survive and/or accumulate in peripheral lymphoid tissues as previously reported (3). Because this ratio is similar to that of MP vs. naïve CD4^+^ T lymphocytes present in steady state (**Figure 1A**, WT), this co-transfer system enables to compare the capacities of these two types of CD4^+^ T cells to give rise to new MP cells on a per population basis in the absence of Tregs.

When donor cells were analyzed on day 7, a small fraction of CD45.1 naïve cells acquired a memory phenotype while CD45.2 MP cells remained MP in WT hosts as we previously reported (3) (**Figure 1E**, WT recipient), and importantly, both MP populations derived from CD45.1 and CD45.2 donor cells expanded by Treg depletion (**Figure 1E**, Foxp3-DTR recipient). These data indicate that 7 days after Treg depletion, both naïve and MP CD4^+^ T lymphocytes can equally contribute to new MP cell generation on a per population basis.

We hypothesized that naïve and MP lymphocytes differentially generate new MP cells at different time points upon Treg depletion. To test this, we labeled CD45.1 naïve and CD45.2 MP donor cells with CFSE and performed the same experiments as described in **Figure 1C**. When analyzed in DT-treated Foxp3-DTR hosts at different time points, CD45.2 donor cells more efficiently provided CFSE^-^ population at early time points (on days 3 and 5) whereas at a later time point (on day 10), CD45.1 donor cells gave rise to more CFSE^−^ cells (**Figure 1F**). In consistent, when composition of CFSE^-^ population among total donor cells was analyzed, CD45.2 and CD45.1 cells were dominant at early and later time points, respectively (**Figure 1G**). By contrast, in DT-treated WT hosts, proliferation of CD45.1 and CD45.2 donor cells was if any minimal (**Supplementary Figure 3**). These findings demonstrate that on a per cell basis, MP and naïve CD4^+^ T lymphocytes preferentially generate rapidly proliferating cells at early and later time points, respectively, post Treg depletion.

In the above experiments, CFSE^+^ and CFSE^−^ populations were almost exclusively Ki67^−^ and Ki67^+^, respectively, in both CD45.1 and CD45.2 donor cells (**Figure 1H**), indicating that Ki67 is a useful marker for distinguishing proliferating from undivided donor cells in this experimental setting. Based on this finding, we further examined expression levels of CD44 and CD62L in dividing vs. non-dividing donor cells. As shown in **Figure 1I**, Ki67^+^ CD45.1 donor cells acquired a memory phenotype whereas their Ki67^−^ counterparts remained naïve. In the case of CD45.2 donor cells, both Ki67^+^ and Ki67^−^ fractions maintained their original memory phenotype. Taken together, these data suggest that Tregs inhibit MP cell generation from preexisting naïve and MP precursors with different kinetics in steady state.

### MP as compared to naïve cells more efficiently generate type 1 responses at an early time point post Treg depletion

**Figure 2:**
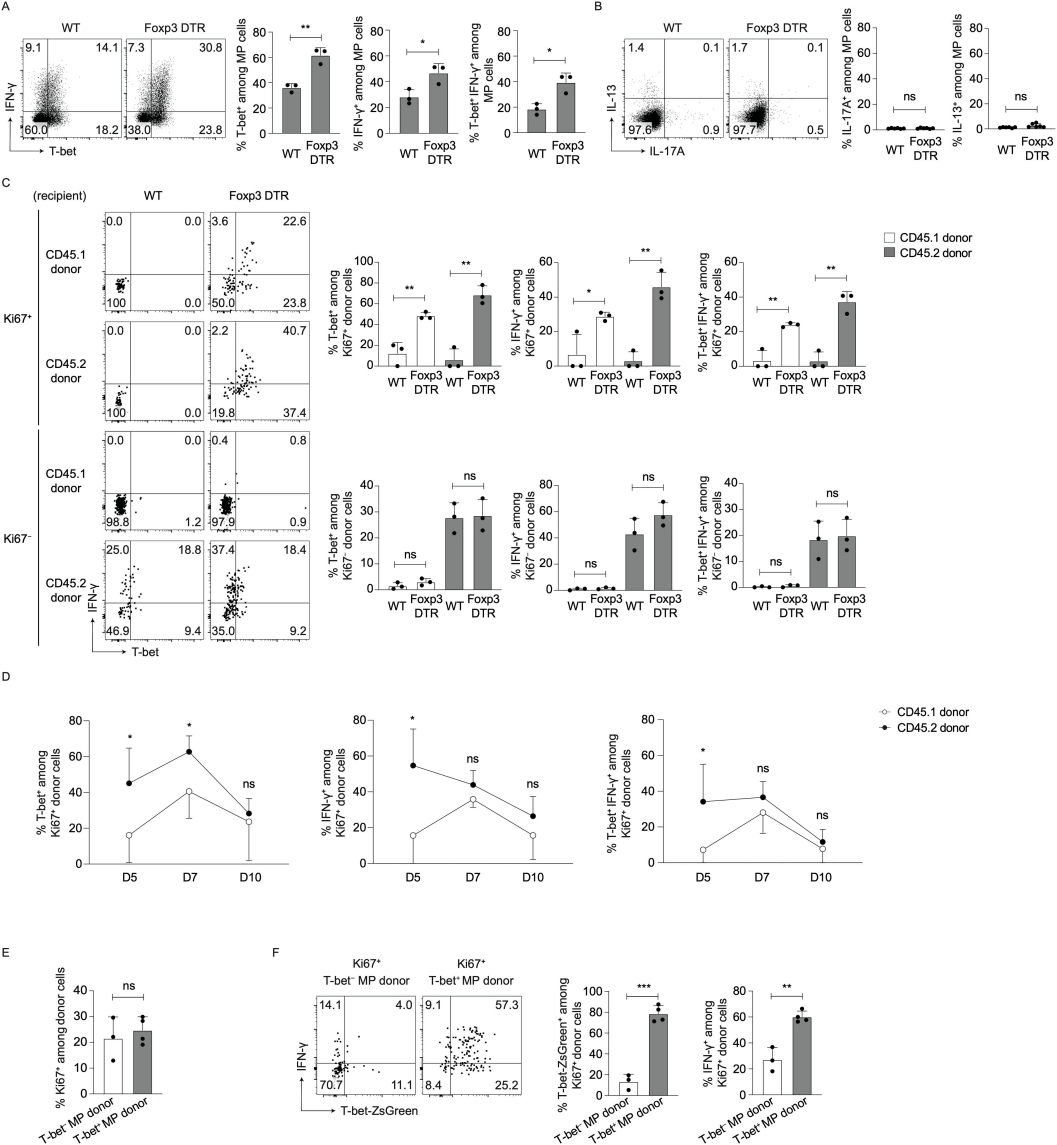
MP cells express higher levels of T-bet and IFN-γ after Treg depletion. **(A, B)** Type 1 differentiation of MP cells is promoted by Treg depletion. Foxp3-DTR and WT mice were injected with DT and splenocytes analyzed on day 7. **(A)** The representative dot plots display T-bet and IFN-γ expression in splenic MP cells while the bar graphs show the frequency (mean ± SD) of T-bet^+^, IFN-γ^+^, and T-bet^+^ IFN-γ^+^ cells among total MP cells from each group (n=3). Data are representative of two independent experiments. **(B)** Representative dot plots showing IL-13 and IL-17A expression in MP cells together with bar graphs indicating the frequency (mean ± SD) of IL-13^+^ and IL-17A^+^ fractions among MP cells (n=6). Data are pooled from two independent experiments. **(C)** Both MP and naïve cells upregulate T-bet and IFN-γ levels through proliferation in the absence of Tregs. CD45.1 naïve and CD45.2 MP cells were co-transferred into CD45.1/2 WT or Foxp3-DTR animals that were subsequently subjected to DT treatment as described in **Figure 1C**, and T-bet and IFN-γ expression in Ki67^+^ and Ki67^-^ donor cells analyzed on day 7. The dot plots display T-bet and IFN-γ expression in the indicated donor cell subpopulations whereas the bar graphs show the frequency (mean ± SD) of T-bet^+^, IFN-γ^+^, and T-bet^+^ IFN-γ^+^ cells among the same subpopulations (n=3). Data are representative of two independent experiments performed. **(D)** Proliferating MP cells more efficiently generate Th1 responses than do naïve cells at early time points post Treg depletion. In the above transfer experiments, T-bet and IFN-γ expression in Ki67^+^ donor cells was analyzed on days 5, 7, and 10. The graphs show the frequency (mean ± SD) of T-bet^+^, IFN-γ^+^, and T-bet^+^ IFN-γ^+^ fractions among donor populations at different time points (n=5). Data are pooled from two independent experiments. **(E, F)** Some T-bet^−^ MP cells acquire a Th1-like phenotype upon Treg depletion whereas the majority of their T-bet^+^ counterparts remain Th1. T-bet^+^ and T-bet^−^ MP cells sorted from CD45.2 T-bet-ZsGreen reporter mice were separately transferred into DT-treated CD45.1/2 Foxp3-DTR recipients, and expression of Ki67, T-bet-ZsGreen, and IFN-γ in donor cells analyzed on day 7. **(E)** The graph shows the frequency (mean ± SD) of Ki67^+^ cells among each donor population (n=3-4). **(F)** Representative dot plots depicting T-bet-ZsGreen and IFN-γ expression in Ki67^+^ donor cells from each group together with bar graphs showing the frequency (mean ± SD) of T-bet-ZsGreen^+^ and IFN-γ^+^ cells among the same donor cells (n=3-4). Data are representative of two independent experiments. * *p*<0.05, ** *p*<0.01, *** *p*<0.001, ns: not significant.

Next, we sought to determine whether Tregs influence Th1/2/17 differentiation of MP and naïve CD4^+^ T lymphocytes. To do so, we examined expression of T-bet and IFN-γ in MP cells from DT-treated WT and Foxp3-DTR mice. Seven days after DT treatment, MP cells in the latter animals expressed higher levels of T-bet and IFN-γ than did those from the former (**Figure 2A**). Regarding Th2/17 differentiation, neither IL-13 nor IL-17A levels were upregulated by Treg depletion (**Figure 2B**). Thus, Treg depletion mainly promotes type 1 responses of MP cells.

To determine which populations, proliferating vs. non-dividing cells derived from preexisting MP vs. naïve CD4^+^ T lymphocytes, contribute to the above upregulation of Th1 responses, we transferred a mixture of CD45.1 naïve and CD45.2 MP cells into CD45.1/2 Foxp3-DTR mice as described in **Figure 1C** and examined T-bet and IFN-γ expression in Ki67^+^ and Ki67^-^ donor cells 7 days later. As shown in **Figure 2C**, Ki67^+^ cells of both CD45.1 and CD45.2 origins expressed higher levels of T-bet and IFN-γ in Foxp3-DTR vs. WT recipient mice whereas no difference was detected in their Ki67^-^ counterparts. To further determine relative contribution of proliferating CD45.2 vs. CD45.1 donor cells to Th1 differentiation at early and later time points post Treg depletion, we examined T-bet and IFN-γ expression in donor cells at different time points. Ki67^+^ CD45.2 donor cells generated more T-bet^+^ IFN-γ^+^ cells than did their CD45.1 counterparts on day 5, the difference of which became undetectable by day 10 (**Figure 2D**). Together these results demonstrate that proliferating MP CD4^+^ T lymphocytes newly generated from preexisting MP as compared to naïve cells exhibit greater degree of Th1 differentiation at an early time point after Treg depletion.

In steady state, MP cells comprise T-bet^−^ and T-bet^+^ subsets (8). To determine which T cell subpopulation contributes to the above Th1 responses, we sorted for T-bet^−^ and T-bet^+^ MP cells from CD45.2 T-bet-ZsGreen reporter mice and separately transferred into CD45.1/2 Foxp3-DTR mice that subsequently received DT for 7 days. As shown in **Figure 2E**, T-bet^−^ and T-bet^+^ MP donor cells proliferated to the same extent, and some T-bet^−^ donor cells differentiated into Th1 whereas the T-bet^+^ donor population largely maintained its T-bet/IFN-γ expression levels (**Figure 2F**). Thus, both preexisting T-bet^−^ and T-bet^+^ MP cells can proliferate and contribute to new Th1 MP cell development in the absence of Tregs.

### MP cell expansion induced by Treg depletion requires TCR – MHC II interactions

**Figure 3:**
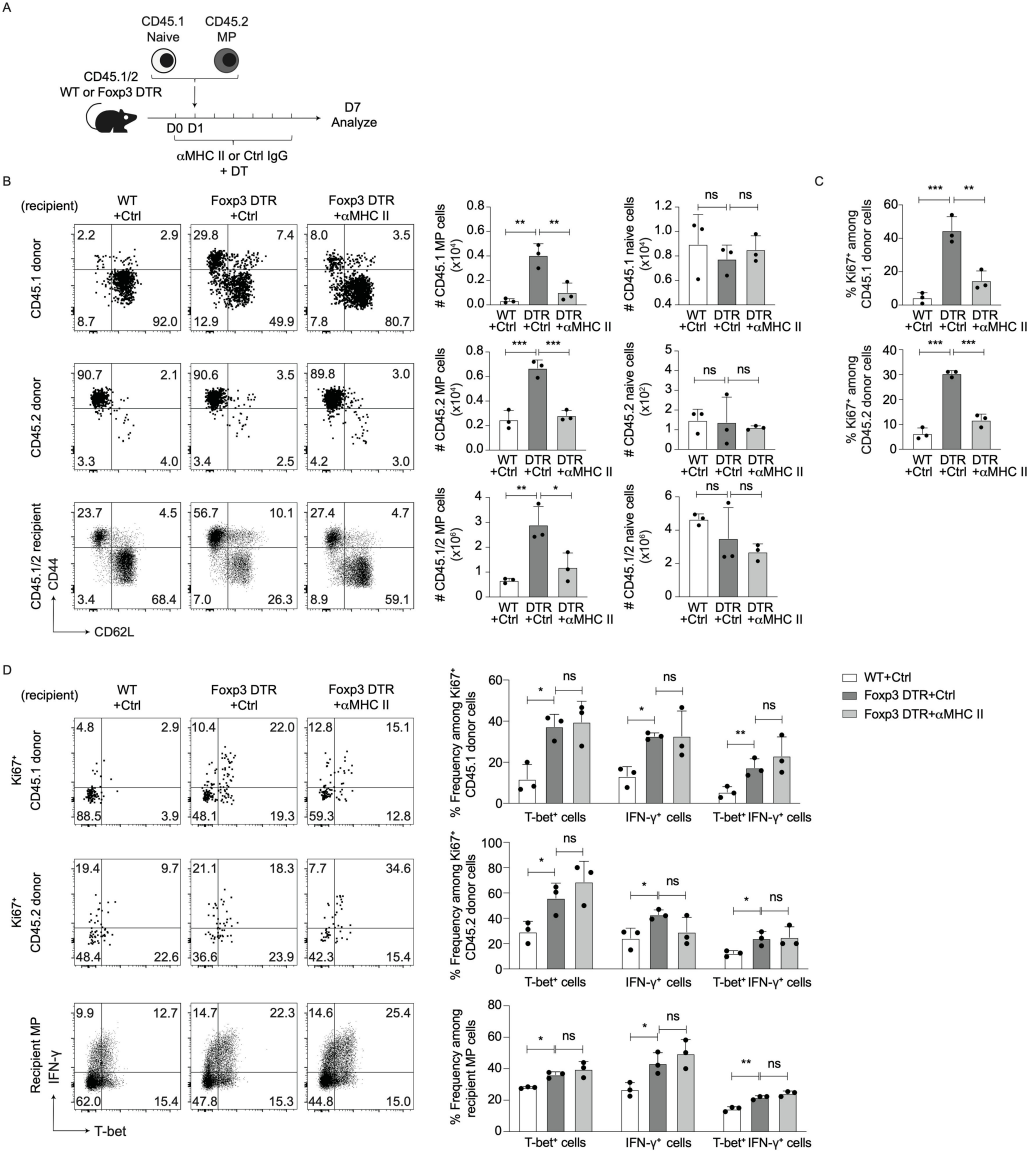
TCR signaling is required for MP cell expansion triggered by Treg depletion. **(A–C)** Treg depletion-driven expansion of MP cells is dependent on TCR signaling. **(A)** Experimental design. DT-treated CD45.1/2 WT and Foxp3-DTR mice received CD45.2 MP and CD45.1 naïve CD4^+^ T cells and were subsequently injected anti-MHC II mAb or control IgG every other day. Donor as well as recipient cells were analyzed on day 7. **(B)** The representative dot plots display CD44 vs. CD62L expression on CD45.1 and CD45.2 donor as well as CD45.1/2 recipient cells while the bar graphs show the absolute number (mean ± SD) of MP and naïve cells in the indicated cell populations (n=3). **(C)** The bar graphs indicate the Ki67^+^ fraction (mean ± SD) among donor cell populations (n=3). Data are representative of two independent experiments. **(D)** Blockade of MHC II - TCR interactions does not significantly reduce T-bet^+^ or IFN-γ^+^ fractions in MP cells of DT-treated Foxp3-DTR mice. The representative dot plots display T-bet and IFN-γ expression in Ki67^+^ donor as well as total recipient MP cells from each group whereas the bar graphs show the frequency (mean ± SD) of T-bet^+^, IFN-γ^+^, and T-bet^+^ IFN-γ^+^ fractions among the indicated cell populations (n=3). Data are representative of two independent experiments. * *p*<0.05, ** *p*<0.01, *** *p*<0.001, ns: not significant.

We next addressed the molecular mechanisms responsible for MP cell proliferation and differentiation in the absence of Tregs. To ask if MP cell activation is dependent on Ag recognition, we co-transferred CD45.1 naïve and CD45.2 MP cells into DT-treated CD45.1/2 WT and Foxp3-DTR mice that were subsequently injected with anti-MHC II monoclonal antibody (mAb) or control IgG for 7 days (**Figure 3A**). Treg depletion induced a marked increase in the number of MP but not naïve cells of both donor and recipient origins, and this MP cell increment was abolished by treatment with anti-MHC II mAb (**Figure 3B**). In consistent, administration of anti-MHC II mAb significantly inhibited proliferation of both CD45.1 and CD45.2 donor cells (**Figure 3C**). Thus, TCR signaling plays an essential role in MP cell generation from both naïve and MP precursors.

To examine the role for TCR signaling in Treg depletion-triggered Th1 responses, T-bet and IFN-γ expression was examined in the above experiments. As shown in **Figure 3D**, blockade of MHC II did not significantly reduce T-bet or IFN-γ levels in Ki67^+^ donor as well as recipient MP cells, arguing that TCR signaling does not seem to significantly promote type 1 responses of naïve or MP cells in the absence of Tregs.

### MP cell proliferation driven by Treg depletion is dependent on CD28 signaling

**Figure 4:**
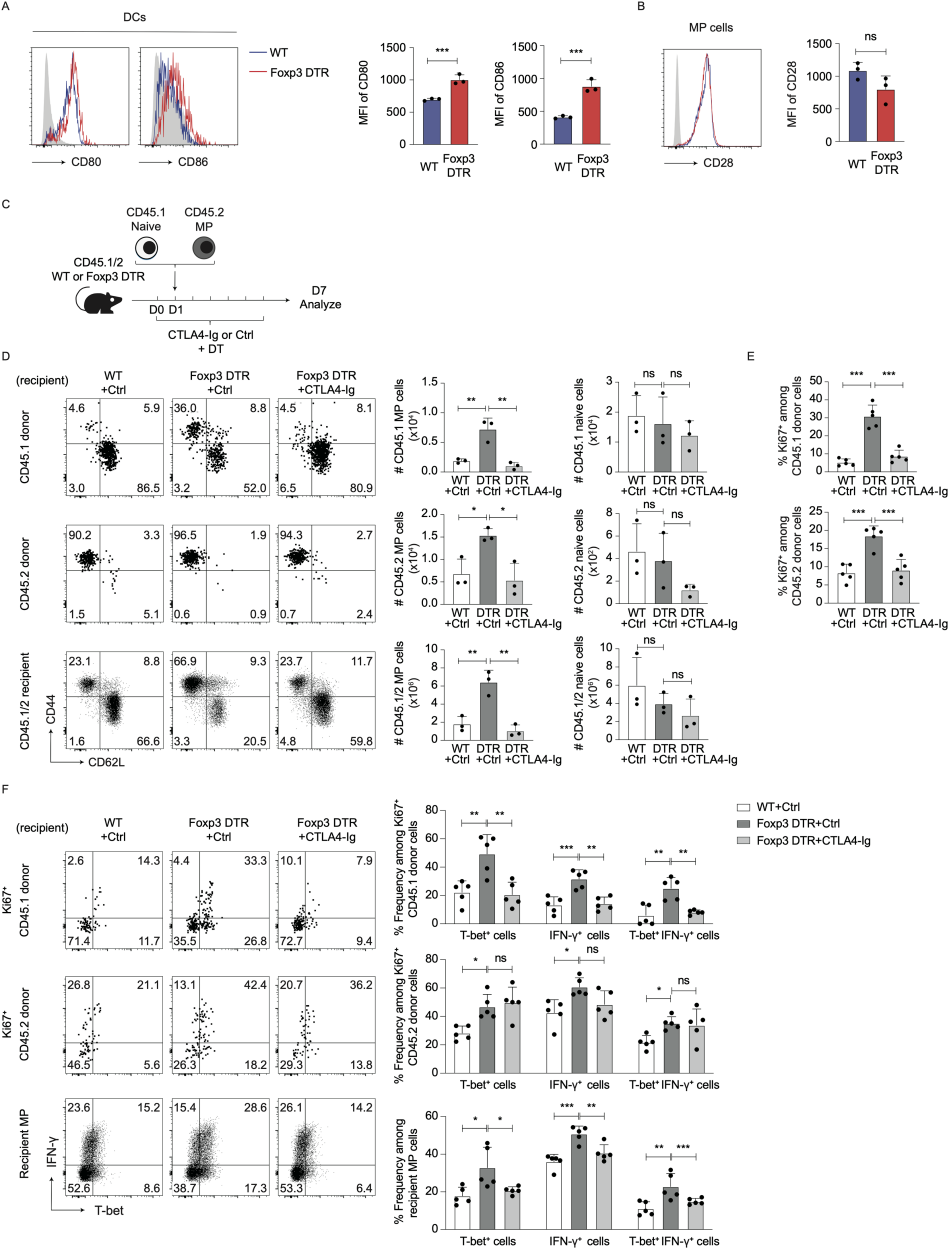
Treg depletion-driven MP cell proliferation is induced by CD28 signaling. **(A, B)** Treg depletion induces upregulation of CD80/86 on DCs without altering CD28 levels on MP cells. Foxp3-DTR and WT mice received DT for 7 consecutive days, and splenic DCs and MP cells analyzed for their CD80, CD86, and CD28 expression. Representative histograms display **(A)** CD80/86 on DCs and **(B)** CD28 on MP cells whereas the bar graphs show the mean fluorescence intensity (MFI) (mean ± SD) of the indicated molecules from each group (n=3). Filled histograms show negative control staining. Data are representative of two independent experiments performed. **(C–E)** CTLA4-Ig treatment inhibits Treg depletion-induced proliferation of MP and naïve donor cells. **(C)** Experimental design. MP and naïve CD4^+^ T cells were sorted from CD45.2 and CD45.1 mice, respectively, and transferred into DT-treated CD45.1/2 Foxp3-DTR mice that were subsequently administered CTLA4-Ig or PBS every other day. Donor as well as recipient cells were analyzed on day 7. **(D)** The representative plots display CD44 vs. CD62L expression on CD45.1 and CD45.2 donor as well as CD45.1/2 recipient cells whereas the bar graphs show the absolute number (mean ± SD) of MP and naïve cells in each donor/recipient cell population (n=3). **(E)** The bar graphs indicate the frequency (mean ± SD) of Ki67^+^ cells among donor cell populations (n=5). Data shown are **(D)** representative of and **(E)** pooled from two independent experiments performed. **(F)** CTLA4-Ig treatment significantly lowers T-bet^+^ and IFN-γ^+^ fractions among MP cells derived from naïve but not MP donor populations in DT-treated Foxp3-DTR mice. The representative dot plots display T-bet and IFN-γ expression in Ki67^+^ donor and total recipient MP populations whereas the bar graphs show the frequency (mean ± SD) of T-bet^+^, IFN-γ^+^, and T-bet^+^ IFN-γ^+^ cells among the indicated cell populations from each group (n=5). Data are pooled from two independent experiments. * *p*<0.05, ** *p*<0.01, *** *p*<0.001, ns: not significant.

Tregs can inhibit T cell immune responses by capturing CD80 and CD86 from DCs (14). Indeed, in the present study Treg depletion significantly upregulated CD80 and CD86 levels on DCs without altering CD28 expression in MP cells (**Figures 4A, B**), suggesting potential involvement of CD28 signaling in hyperactivation of MP cells driven by Treg depletion.

To determine if CD28 signals are required for the MP cell responses, we made use of CTLA4-Ig that blocks CD80 and to a lesser extent CD86 (18, 19). Specifically, we co-transferred CD45.1 naïve and CD45.2 MP cells into DT-treated CD45.1/2 WT or Foxp3-DTR recipients that were subsequently subjected to CTLA4-Ig treatment (**Figure 4C**). The MP cell expansion induced by Treg depletion was almost completely blocked by CTLA4-Ig treatment (**Figure 4D**). In consistent, the same treatment significantly reduced Ki67 expression in CD45.1 and CD45.2 donor cells (**Figure 4E**). These results argue that Treg depletion triggers both new MP generation from naïve precursors and further proliferation of preexisting MP cells in a CD28 signaling-dependent manner.

We also examined whether CD28 signals are involved in Treg depletion-driven type 1 responses of MP cells. As shown in **Figure 4F**, CTLA4-Ig treatment did not affect T-bet or IFN-γ levels in Ki67^+^ CD45.2 donor cells whereas in Ki67^+^ CD45.1 donor as well as recipient MP cell populations, the same treatment significantly inhibited expression of these molecules. Thus, type 1 responses of preexisting MP cells driven by Treg depletion are less dependent on CD28 signaling as compared to those of naïve CD4^+^ T lymphocytes.

### Treg depletion-driven Th1 responses of MP cells are mediated by IL-2 signaling

Tregs can suppress T cell immune responses by IL-2 consumption (15, 16). This notion led us to hypothesize that IL-2 is involved in the MP cell activation in our present study as well. Indeed, serum IL-2 concentration was heightened upon Treg depletion (**Supplementary Figure 4A**), supporting this hypothesis. Regarding the source of the same cytokine, MP but not naïve CD4^+^ T lymphocytes tonically expressed IL-2 in IL-2-eGFP reporter mice (**Supplementary Figure 4B**) as previously suggested (15, 16). Consistently, low levels of IL-2 protein were detected in MP and to a lesser extent naïve CD4^+^ T cells in steady-state WT animals (**Supplementary Figure 4C**). Although their IL-2 production was unaltered by Treg depletion on a per cell basis (**Supplementary Figure 4D**), the increment in total MP cell number (**Figure 1A**) and/or augmented IL-2 production by other types of cells including CD8^+^ T lymphocytes as well as NK cells (20) may account for the increased serum IL-2 levels in the absence of Tregs.

**Figure 5:**
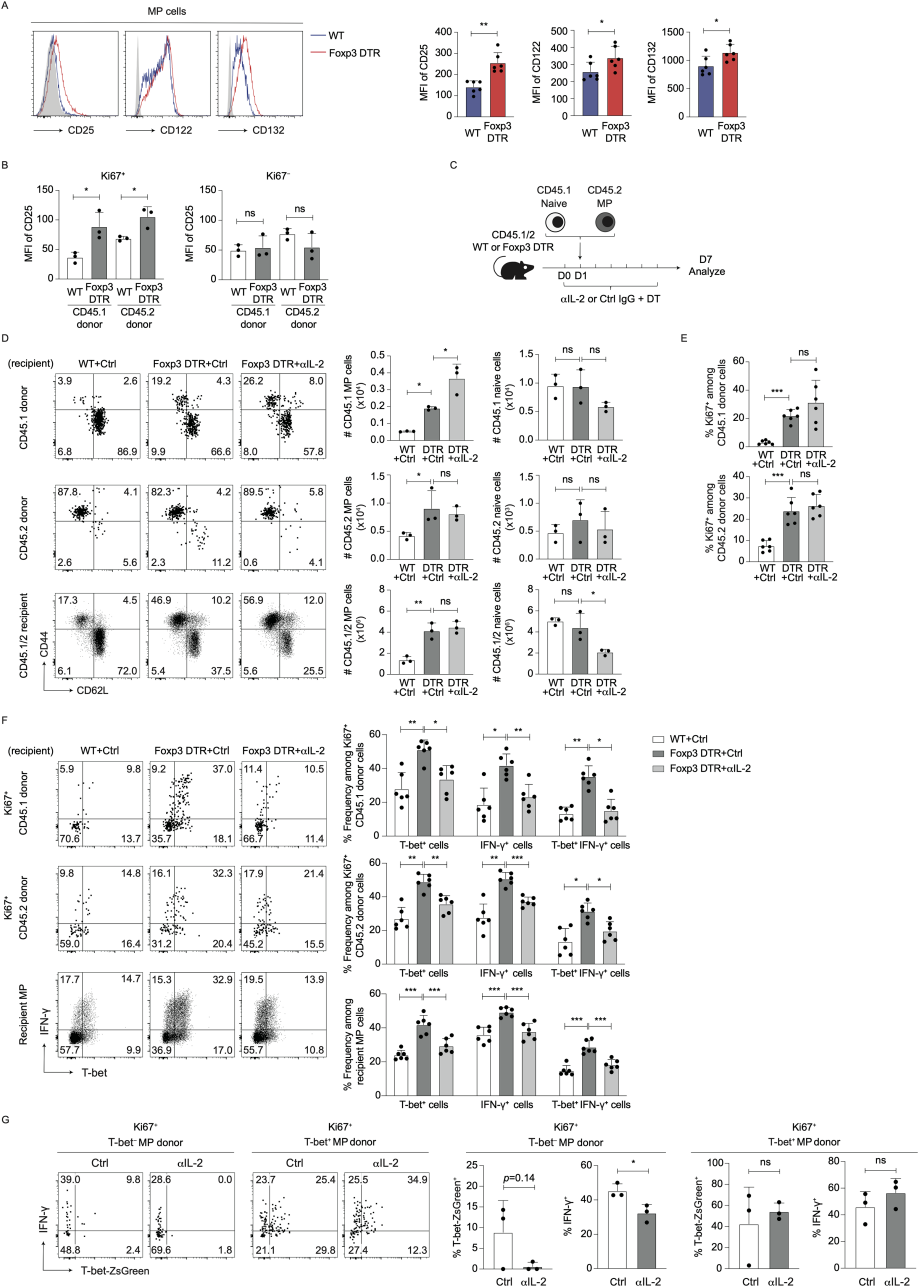
IL-2 signaling enhances Th1 responses of T-bet^-^ MP cells post Treg depletion. **(A)** CD25, CD122, and CD132 expression levels in MP cells are upregulated by acute Treg depletion. The representative histograms display CD25, CD122, and CD132 in MP cells while the bar graphs show the MFI (mean ± SD) of the indicated molecules from each group (n=6). Filled histograms show negative control staining. Data are pooled from two independent experiments performed. **(B)** Both preexisting MP and naïve cells upregulate CD25 expression via proliferation upon Treg depletion. In experiments described in **Figure 1C**, CD25 expression levels on CD45.1 as well as CD45.2 donor cells were analyzed on day 7. The bar graphs indicate the MFI (mean ± SD) of CD25 among Ki67^+^ and Ki67^−^ donor cells from each group (n=3). Data are representative of two independent experiments. **(C–E)** Blockade of IL-2 does not inhibit proliferation of MP cells in the absence of Tregs. **(C)** Experimental design. Sorted CD45.2 MP and CD45.1 naïve CD4^+^ T cells were transferred to DT-injected CD45.1/2 Foxp3-DTR mice that were subsequently subjected to treatment with anti-IL-2 mAb or control IgG every other day. Donor as well as recipient cells were analyzed on day 7. **(D)** The representative dot plots show expression of CD44 and CD62L in CD45.1 and CD45.2 donor as well as CD45.1/2 recipient cells while the bar graphs show the absolute number (mean ± SD) of MP and naïve cells in each donor/recipient cell population (n=3). **(E)** Bar graphs showing Ki67 expression (mean ± SD) in the indicated donor cell populations are displayed (n=6). Data shown are **(D)** representative of and **(E)** pooled from two independent experiments performed. **(F)** Anti-IL-2 mAb treatment significantly inhibits T-bet and IFN-γ expression levels in donor and recipient MP cells in Foxp3-DTR mice treated with DT. The representative dot plots show expression of T-bet and IFN-γ in Ki67^+^ donor as well as total recipient MP cells whereas the bar graphs indicate the positive fractions (mean ± SD) of T-bet and IFN-γ in the same populations from each group (n=6). Data are pooled from two independent experiments. **(G)** IL-2 promotes Treg ablation-driven Th1 differentiation of T-bet^−^ MP cells. T-bet^−^ and T-bet^+^ MP cells were sorted from CD45.2 T-bet-ZsGreen reporter mice and separately transferred into DT-treated CD45.1/2 Foxp3-DTR mice that subsequently received anti-IL-2 mAb or control IgG. T-bet-ZsGreen and IFN-γ expression in donor cells was analyzed on day 7. Representative dot plots depicting T-bet-ZsGreen and IFN-γ expression in Ki67^+^ donor cells from each group together with bar graphs showing the frequency (mean ± SD) of T-bet-ZsGreen^+^ and IFN-γ^+^ cells among the same donor cell populations are displayed (n=3). * *p*<0.05, ** *p*<0.01, *** *p*<0.001, ns: not significant.

We also compared expression of CD25 (IL-2Rα), CD122 (IL-2Rβ), and CD132 (IL-2Rγ) on MP cells between DT-treated WT and Foxp3-DTR mice and detected upregulated expression of these receptors in the latter hosts (**Figure 5A**). To ask which population(s), preexisting naïve or MP cells, contribute to generation of CD25^hi^ CD44^hi^ CD62L^lo^ CD4^+^ T cells in the absence of Tregs, we performed the same transfer experiments as described in **Figure 1C** and analyzed CD25 levels in CD45.1 and CD45.2 donor cells. As shown in **Figure 5B**, Ki67^+^ donor cells of both CD45.1 and CD45.2 origins upregulated their CD25 levels after Treg depletion. Thus, MP cells newly generated from both preexisting naïve and MP cells upregulate their CD25 levels when Tregs are depleted.

To seek the roles for IL-2 signaling in MP cell proliferation and differentiation in the absence of Tregs, we further co-transferred CD45.1 naïve and CD45.2 MP cells into DT-treated CD45.1/2 Foxp3-DTR or WT hosts that were then administered anti-IL-2 mAb or control IgG (**Figure 5C**). On day 7, blockade of IL-2 had no inhibitory effects on Treg depletion-induced MP cell expansion in CD45.1, CD45.2, or CD45.1/2 populations (**Figure 5D**). Consistently, anti-IL-2 mAb did not inhibit proliferation of CD45.1 or CD45.2 donor cells (**Figure 5E**). Hence, IL-2 signaling is not required for Treg depletion-induced MP cell proliferation.

We also addressed whether type 1 responses of MP cells triggered by Treg ablation is IL-2-dependent. As shown in **Figure 5F**, blockade of IL-2 significantly inhibited T-bet and IFN-γ expression levels in Ki67^+^ donor as well as recipient MP cells. These data argue that IL-2 signaling mediates Treg depletion-triggered Th1 responses of MP as well as naïve CD4^+^ T cells.

The forementioned results in **Figure 2F** suggest that both preexisting T-bet^-^ and T-bet^+^ MP subpopulations can contribute to type 1 MP cell responses driven by Treg depletion. To determine which MP subpopulation, T-bet^−^ or T-bet^+^, IL-2 acts on, we separately transferred T-bet^−^ and T-bet^+^ MP cells into DT-treated Foxp3-DTR mice that were then injected with anti-IL-2 mAb or control IgG. Blockade of IL-2 reduced IFN-γ expression in T-bet^−^ MP donor cells without affecting their T-bet^+^ counterparts (**Figure 5G**). These data argue that IL-2 plays an important role in acquisition of Th1-like phenotype in T-bet^−^ MP cells but is dispensable for maintenance of the same phenotype in terminally differentiated T-bet^+^ MP cells.

### Early MP cell activation leads to multi-organ inflammation in the absence of Tregs

**Figure 6:**
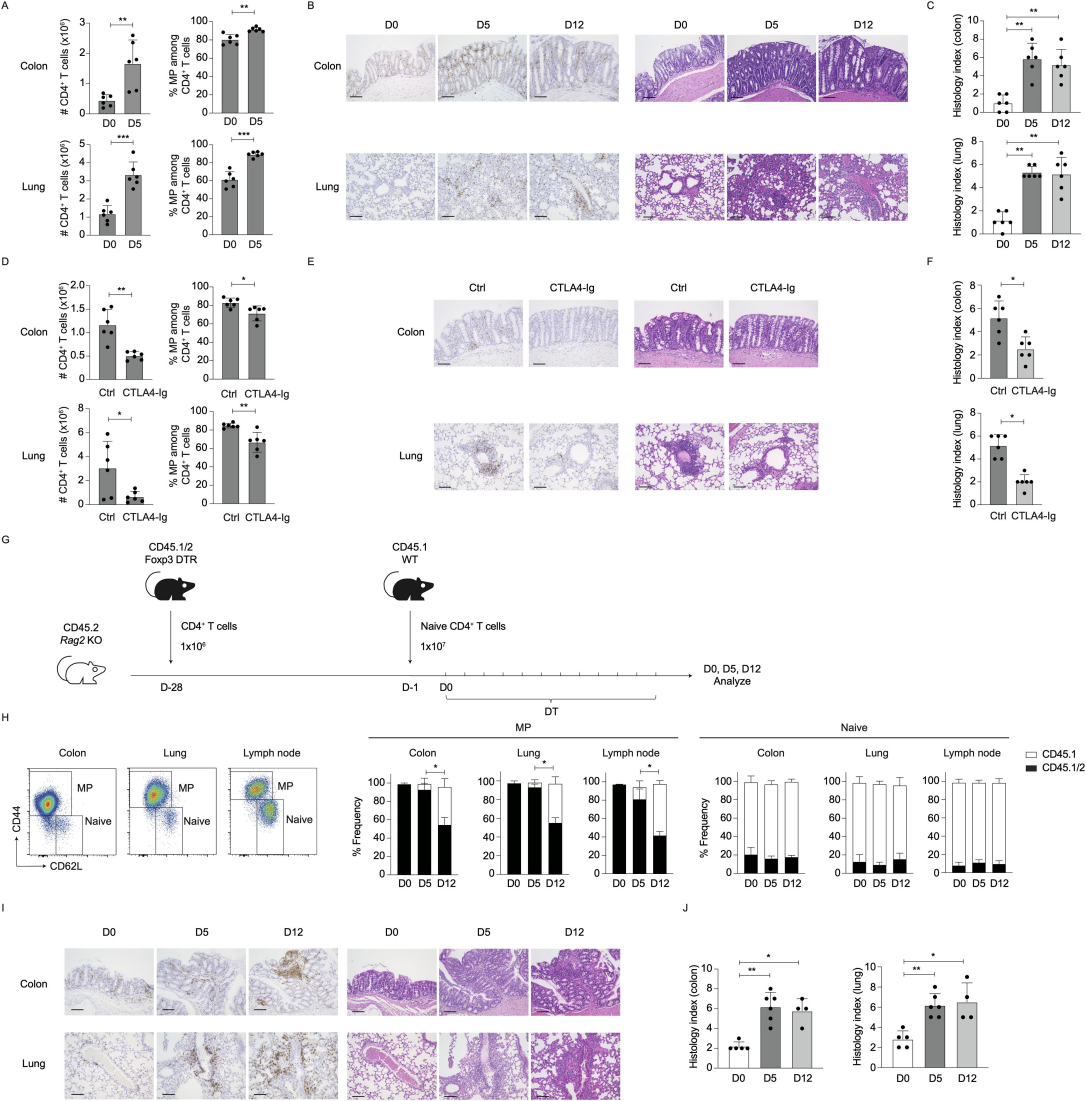
Preexisting MP cells critically contribute to early inflammatory responses in non-lymphoid organs upon Treg depletion. **(A–C)** CD4^+^ T lymphocytes accumulate in the colon and lungs at an early time point post Treg depletion. Foxp3-DTR and WT mice received DT for the indicated period of time. **(A)** The bar graphs show the number (mean ± SD) of CD4^+^ T cells present in the indicated organs and their MP cell fraction (mean ± SD) on days 0 and 5 (n=6). **(B, C)** Representative images of H&E and CD4-directed immunohistochemical staining together with bar graphs indicating histological scores (mean ± SD) of colonic and lung inflammation at the indicated time points are shown (n=6). Data are pooled from two independent experiments performed. **(D–F)** CTLA4-Ig treatment inhibits early CD4^+^ T cell accumulation in extra-lymphoid organs. In the above experiments, CTLA4-Ig or control PBS were injected. **(D)** The bar graphs depict the absolute number (mean ± SD) of CD4^+^ T cells accumulating in the indicated organs and the frequency (mean ± SD) of MP cells among the same T cell populations on day 5 (n=6). **(E, F)** Representative images of H&E and CD4-directed immunohistochemical staining together with bar graphs indicating histological scores (mean ± SD) of inflammation on day 5 are displayed (n=6). Data are pooled from two independent experiments performed. **(G–J)** Preexisting MP cells constitute the dominant CD4^+^ T cell population accumulating in the colon and lungs at an early time point post Treg depletion. **(G)** Experimental design. 1×10^6^ total CD4^+^ T cells from CD45.1/2 Foxp3-DTR mice (D−28) and 1×10^7^ naïve CD4^+^ T cells from CD45.1 mice (D−1) were sequentially transferred into CD45.2 *Rag2* KO mice that were subsequently treated with DT every day starting on day 0. MP and naïve CD4^+^ T cells were analyzed for CD45.1 and CD45.2 expression on days 0, 5, and 12. **(H)** The bar graphs show the frequency (mean ± SD) of CD45.1 and CD45.1/2 subpopulations among total naïve and MP cells in the indicated organs (n=4-6). Representative dot plots displaying CD44 and CD62L expression in CD4^+^ T cells in the indicated organs on day 0 are also included. **(I, J)** Representative images displaying H&E and CD4-directed immunohistochemical staining as well as bar graphs indicating histological scores (mean ± S.D.) of colitis and lung inflammation are shown (n=4-6). Data are pooled from two independent experiments. Scale bars: 100 μm. * *p*<0.05, ** *p*<0.01, *** *p*<0.001.

Because MP CD4^+^ T cells exhibit rapid proliferation as well as Th1 differentiation in lymphoid tissues at an early time point post Treg depletion (**Figures 1**, **2**), we hypothesized that the same T lymphocytes can induce inflammation in extra-lymphoid organs as well. To test this, we analyzed CD4^+^ T lymphocytes in the colon and lungs 5 days after Treg ablation. As shown in **Figure 6A**, CD4^+^ T cells and especially their MP fractions expanded in both organs. In consistent, histological analyses revealed that CD4^+^ cells infiltrated into lamina propria of the colon, interstitial potions of lungs and kidneys, and Glisson’s sheaths of the liver (**Figure 6B**; **Supplementary Figure 5A**). In parallel, histological inflammation scores in the colon and lungs were significantly higher on days 5 and 12 (**Figure 6C**). These data show early CD4^+^ T cell accumulation in non-lymphoid organs in Treg-depleted environments.

Based on the observations that CD28 but not IL-2 signaling is essential for splenic MP cell expansion in the absence of Tregs (**Figures 4**, **5**), we examined involvement of the same signals in CD4^+^ T cell orchestration to extra-lymphoid organs. CTLA4-Ig treatment significantly inhibited CD4^+^ MP cell accumulation and histological inflammation in the colon and lungs on day 5 post Treg depletion (**Figures 6D–F**). On the other hand, blockade of IL-2 did not affect the number of CD4^+^ T cells or their MP fractions in these organs (**Supplementary Figure 5B**). Thus, Treg depletion-induced early T cell accumulation in extra-lymphoid organs is dependent on CD28 signaling.

Finally, we asked which population, preexisting MP or naïve cells, contributes to the above early CD4^+^ MP cell accumulation in the colon and lungs. We previously established an experimental system where MP and naïve CD4^+^ T cells can be marked with different congenic markers by transferring total CD4^+^ T cells from CD45.1/2 mice into *Rag2* KO animals that subsequently receive a large number of purified naïve CD4^+^ T cells of CD45.1 origins 4 weeks later (3). In this experimental setting, the first cohort of donor cells robustly proliferates to give rise to CD45.1/2 MP cells and Tregs whereas the second cohort remains CD45.1 naïve in the environment where the niche for MP cells and Tregs are occupied by the former donor population (21).

Based on the above protocol, we transferred CD45.1/2 total CD4^+^ T cells obtained from Foxp3-DTR mice into *Rag2*-deficient animals (**Figure 6G**, D-28), followed by CD45.1 naïve cell transfer 4 weeks later (D-1). The host mice were then subjected to DT treatment to deplete CD45.1/2 Tregs. On day 0, MP and naïve cells were dominated by CD45.1/2 and CD45.1 populations, respectively, in the colon, lungs, and lymph nodes (**Figure 6H**, D0), validating the experimental system. When analyzed at later time points, the majority of MP cell population on day 5 was CD45.1/2 while that on day 12 contained a significant fraction of CD45.1 cells (**Figure 6H**, MP). Here, naïve cells were CD45.1 at all times (**Figure 6H**, Naïve). Furthermore, on day 5, a majority of CD4^+^ T cells that are dominated by preexisting MP-derived cells (**Figure 6H**) resided in colonic lamina propria and interstitial part of lungs, the response of which was accompanied by augmented histological inflammation (**Figures 6I, J**). Taken together, at an early time point post Treg depletion, preexisting MP but not naïve CD4^+^ T cells play a dominant role in the induction of inflammatory responses in non-lymphoid organs.

## Discussion

In the present study, we have found that both preexisting MP and naïve CD4^+^ T lymphocytes robustly proliferate and generate Th1 responses when Tregs are acutely depleted. MP cell activation occurs at an early time point post Treg depletion whereas naïve cells play a dominant role at a later stage. Moreover, our data argue that MP cell proliferation and Th1 responses are differentially regulated by TCR, CD28, and IL-2 signaling; the former process requires TCR and CD28 signaling while the latter is dependent on IL-2. Furthermore, our findings suggest that such early MP cell activation can lead to multi-organ inflammation. Together these observations reveal the essential role for Tregs in MP cell homeostasis in steady state.

CD4^+^ T lymphocytes are strictly regulated by homeostatic mechanisms throughout animal’s life. Thus, when situated under lymphopenic environments, T cells exhibit a proliferative response referred to as “homeostatic proliferation” to recover lymphosufficiency (1, 2). Traditionally, this response has been studied by transferring naïve T lymphocytes into irradiated or *Rag1/2*-deficient mice. In such lymphopenic animals, some of transferred naïve CD4^+^ T lymphocytes rapidly proliferate to convert to MP cells in a manner dependent on Ag recognition as well as co-stimulation provided by APCs (22–27). Once MP cells are generated, these cells themselves exhibit rapid proliferation in lymphopenic environments (28–30), maintaining T cell homeostasis.

Importantly, the above homeostatic proliferation can be driven in physiological, lymphosufficient environments as well. Thus, when naïve CD4^+^ T cells are transferred into lymphoreplete WT hosts, some donor cells proliferate to give rise to MP cells, although at a lower rate (3). In the case of lymphopenia-induced proliferation, naïve cell expansion is known to be inhibited by co-transfer of Tregs (31–33), suggesting that Tregs may play an inevitable role in suppression of excess homeostatic proliferation in lymphoreplete conditions. However, it was unclear whether MP CD4^+^ T lymphocytes exhibit proliferative responses when transferred into lymphosufficient hosts, and whether Tregs exert differential suppressive activities on homeostatic proliferation of MP vs. naïve CD4^+^ T lymphocytes in steady state.

In the present study, we transferred naïve and MP cells into WT as well as Treg-depleted mice and examined behaviors of these two types of donor cells. We found that both naïve and MP cells minimally proliferate in WT hosts (**Supplementary Figure 3**), suggesting that the latter cells have a potential to exhibit proliferative responses in lymphosufficient environment in a similar manner to the former lymphocytes. Importantly, a previous report demonstrates slow but persistent proliferation of foreign Ag-specific “conventional” memory CD4^+^ T cells that is characterized by one to two cell division(s) in lymphoreplete hosts (30). Because MP cells did not exhibit such one to two cell division(s) in WT environments (**Supplementary Figure 3**), these two types of T lymphocytes may be different in terms of proliferative behaviors. Indeed, we have recently identified a few markers that are differentially expressed in MP vs. foreign Ag-specific memory cells; i.e., the former cells adopt a CD127^lo-hi^ Sca1^lo-hi^ phenotype whereas the latter cells are CD127^hi^ Sca1^hi^ (34). Given the observation that CD127^hi^ Sca1^hi^ and CD127^lo^ cells homeostatically exhibit slow and fast cell divisions, respectively, as reported in the same study, difference in proliferative behaviors between MP and memory cells in lymphosufficient hosts may reflect that of their constituents in steady state.

Upon acute Treg depletion, MP donor cells rapidly proliferated to give rise to Th1 cells at an early time point whereas these responses were dominated by naïve-derived donor cells at a later stage (**Figures 1**, **2**). What makes such difference between MP and naïve cells? One possibility is that MP cells can proliferate faster in speed than their naïve counterparts. Indeed, a substantial fraction of MP but not naïve cells is in cell cycle in steady state (34), supporting this hypothesis. The other explanation that is mutually non-exclusive to the above is that steady-state MP cells contain a subpopulation which is quickly activated upon Treg depletion. It needs further investigation to determine whether or not this rapidly stimulated subpopulation is equivalent to CD127^lo^ MP cells described above. In either case, these results suggest that Tregs homeostatically inhibit spontaneous activation of MP cells in steady state. Given that MP cells tonically express Ki67 and differentiate into Th1 cells at homeostasis (6–8), Tregs are thought to play an essential role in suppressing inflammatogenic nature of MP cells.

Curiously, rapidly proliferating donor cells were always detected as CFSE^-^ cells, and those showing one to two cell division(s) were hardly seen, even at the earliest time point post Treg depletion (**Figure 1F**). This might be because cells that have just started fast cell division are firmly attached to APCs including DCs and thus it is difficult to analyze such cells by flow cytometry, as suggested by previous literature (27).

Our present data show that in addition to TCR – MHC II interactions, CD28 signaling critically contributes to Treg depletion-induced MP cell expansion. Thus, CD80 and CD86 expression was upregulated on DCs when Tregs were depleted, and CTLA4-Ig treatment inhibited proliferation of MP cells derived from both naïve and MP precursors (**Figure 4**). In this regard, Tregs are well known to highly express CTLA4, which captures CD80 and CD86 from DCs by trans-endocytosis (14, 35). In addition, it has also been reported that CTLA4 on Tregs can suppress CD80 and CD86 gene transcription in DCs via STAT3 signaling (36). It is therefore possible that these machineries are operative in steady state as well to maintain MP cell homeostasis.

Interestingly, IL-2 seems to be dispensable for Treg depletion-mediated MP cell expansion whereas critical for optimal Th1 differentiation of T-bet^−^ MP cells. Given that IL-2 is homeostatically produced by a small fraction of activated CD4^+^ T cells and that the same cytokine is consumed by surrounding Tregs (15, 16), it is likely that upon acute depletion of Tregs, some CD4^+^ T lymphocytes receive heightened levels of IL-2 signaling. In consistent, newly generated, rapidly proliferating MP cells expressed higher levels of IL-2Rα (**Figure 5**), indicative of augmented IL-2 signals CD4^+^ T cells receive in the absence of Tregs. Importantly, we found that this IL-2 signaling is essential for Th1 responses of both naïve and T-bet^−^ MP cells. Because IL-2 can induce *Il12rb2* and *Tbx21* expression (37) and because low levels of functional IL-12 are homeostatically produced by DCs (8), IL-2 consumption by Tregs may be important to avoid excess degree of IL-12-mediated Th1 differentiation of preexisting T-bet^-^ MP cells in steady state.

Why do MP cells need to express low levels of IL-2 at homeostasis at the risk of their overactivation? A possibility lies in maintenance of Tregs. Thus, in steady state, IL-2 expressed by activated CD4^+^ T lymphocytes is consumed by Tregs for their survival (15, 16). In accord with this hypothesis, blockade of IL-2 augmented rather than inhibited MP cell expansion from naïve precursors in DT-treated Foxp3-DTR animals (**Figure 5D**, CD45.1 donor). This increment in the MP fraction may be attributed to by lowered survival and/or suppressive function of Tregs in the absence of sufficient levels of IL-2 given that there are some residual Tregs that recover in number at a later time point post DT treatment (**Supplementary Figure 1**) as previously described (17).

Altogether, our present data reveal that Tregs play distinct roles in MP vs. naïve CD4^+^ T cell homeostasis with different kinetics. Given that MP cells exhibit tonic proliferation in steady state (3, 6, 7) and are rapidly activated to induce multi-organ inflammation post Treg depletion (**Figure 6**), we speculate that MP cells could trigger certain types of autoimmune and/or inflammatory diseases in humans. In this regard, previous studies show that CD4^+^ T lymphocytes with a memory phenotype are present in human fetal spleen and intestine as well as cord blood (38–40). Because the presence of foreign antigens in these organs is thought to be minimal, this T cell population may represent self-reactive MP cells. Furthermore, a recent paper reported that in patients with *FOXP3* mutation, T cells clonally expand to induce tissue-specific inflammation even in sterile conditions (41), suggesting that Treg depletion could cause activation of self-reactive T cells to induce autoimmune disease. If such potentially inflammatogenic, self-reactive MP CD4^+^ T lymphocytes are existent in humans, blockade of their activation may be beneficial as a novel therapeutic approach for autoimmune and/or inflammatory diseases.

## Materials and methods

### Mice

C57BL/6 CD45.2 WT mice were purchased from Japan SLC (Hamamatsu, Japan). *Rag2* ^-/-^ and CD45.1 WT mice were obtained from the breeding stocks at Tohoku University Graduate School of Medicine. T-bet-ZsGreen/Foxp3-RFP double reporter mice were generated by crossing Foxp3-RFP mice (42) with the T-bet-ZsGreen strain (43) and obtained from the National Institute of Allergy and Infectious Diseases (NIAID) contract facility at Taconic Biosciences. Foxp3-DTR-GFP transgenic mice (17) were provided by G. J. Hämmerling (German Cancer Research Center, Heidelberg, Germany). CD45.1/2 Foxp3-DTR animals were generated by crossing CD45.2 Foxp3-DTR mice with the CD45.1 strain. IL-2-eGFP reporter mice (44) were obtained from C. T. Weaver (University of Alabama at Birmingham, Birmingham, AL). All mice were maintained in SPF animal facilities in Tohoku University Graduate School of Medicine and used at the age of 8-12 weeks. The care and handling of the animals used in our studies were in accordance with the animal study protocol approved by the Institutional Committee for the Use and Care of Laboratory Animals of Tohoku University.

### *In vivo* chemical and mAb treatment

To deplete Tregs in Foxp3-DTR mice, DT (Merck, Darmstadt, Germany) was intraperitoneally injected every day (1 μg/20 g body weight). To block TCR, CD28, and IL-2 signaling, anti-MHC II mAb (Y3P), recombinant CTLA4-Ig, and anti-IL-2 mAb (S4B6) (all from Bio X Cell, West Lebanon, NH) were intraperitoneally administered every other day (300 μg/20 g body weight).

### Cell sorting and adoptive transfer

Total CD4^+^ T lymphocytes were obtained from pooled splenocytes using CD4 Microbeads (Miltenyi Biotec, Bergisch Gladbach, Germany). MP and naïve cells were purified by sorting for CD4^+^ CD25^−^ CD44^hi^ CD62L^lo^ and CD4^+^ CD25^−^ CD44^lo^ CD62L^hi^ cells, respectively, using FACS Aria II (BD Biosciences, San Jose, CA). To obtain T-bet^+^ and T-bet^−^ MP cells, CD4^+^ Foxp3-RFP^−^ CD44^hi^ CD62L^lo^ T-bet-ZsGreen^+^ and CD4^+^ Foxp3-RFP^−^ CD44^hi^ CD62L^lo^ T-bet-ZsGreen^−^ populations, respectively, were sorted out. Purity was >97%. In **Figures 1F–H**, sorted cells were labeled with CFSE (Thermo Fisher Scientific, Waltham, MA) following the manufacturer’s protocols. 1×10^6^ cells were intravenously injected into sex-matched recipient mice.

### Flow cytometric analysis

Single cell suspensions were prepared from spleens and lymph nodes and red blood cells lysed in ACK buffer. To detect donor cells in transfer experiments, CD4^+^ cells were further enriched using CD4 Microbeads. To obtain mononuclear cells from colonic lamina propria and lungs, Lamina Propria Dissociation Kit and Multi Tissue Dissociation Kit 1 (both from Miltenyi Biotec), respectively, were utilized following the manufacturer’s protocols. For intracellular detection of IFN-γ, IL-17A, and IL-13, lymphocytes were incubated in RPMI complete media containing PMA (20 ng/mL) and ionomycin (1 μg/mL) (both from Fujifilm Wako, Osaka, Japan) at 37 °C for 4 hours in the presence of Brefeldin A (1 μg/mL, Biolegend, San Diego, CA). In the case of IL-2 detection, cells were incubated in the absence of PMA and ionomycin. Cells were then suspended in PBS supplemented with 2% fatal bovine serum and incubated with CD16/32 mAb for 10 min, followed by incubation with the following mAbs for 20 min on ice: CD3 (17A2), CD4 (RM4-5) (Thermo Fisher Scientific), CD44 (IM7) (BD Biosciences), CD11c (N418), CD19 (6D5), CD25 (PC61), CD28 (37.51), CD45.1 (A20), CD45.2 (104), CD62L (MEL-14), CD80 (16-10A1), CD86 (GL-1), CD122 (5H4), CD132 (TUGm2), anti-I-A/I-E (M5/114.15.2), and anti-NK1.1 (PK136) (Biolegend). To detect intracellular products, cells were further fixed and permeabilized using Foxp3/Transcription Factor Staining Buffer Set for 20 min on ice and stained with mAbs against Foxp3 (FJK-16s), IL-13 (eBio13A), Ki67 (SolA15) (Thermo Fisher Scientific), IFN-γ (XMG1.2) (BD Biosciences), IL-2 (JES6-5H4), and/or IL-17A (TC11-18H10.1) (Biolegend) for 40 min on ice. For T-bet detection, fixed cells were stained with anti-T-bet mAb (O4-46, BD Biosciences) for 2 hours at room temperature. Flow cytometry was performed using LSR Fortessa and the data analyzed with FlowJo software (both from BD Biosciences). Gating strategies are detailed in **Supplementary Figure 6**.

### Cytokine measurement by ELISA

Serum IL-2 levels were measured using mouse IL-2 Quantikine ELISA Kit (R&D Systems, Minneapolis, MN).

### Histological assessment of inflammation

Histological assessment of colon, lungs, kidneys and liver was performed by hematoxylin and eosin (H&E) and immunohistochemical staining. For the latter staining, rabbit CD4 mAb (EPR19514; diluted in 1:2000) (Abcam, Cambridge, UK) and secondary anti-rabbit antibody conjugated with peroxidase (Nichirei Biosciences, Tokyo, Japan) were used to visualize CD4^+^ cells. Histology indexes of colitis and lung inflammation were evaluated as previously reported (45, 46). Briefly, eight histological components were assessed for colon: ‘inflammatory infiltrate’, ‘goblet cell loss’, ‘crypt density’, ‘crypt hyperplasia’, ‘muscle thickness’, ‘submucosal infiltration’, ‘ulcerations’, and ‘crypt abscesses’ and three parameters for lungs: ‘vascular features’, ‘extravascular and alveolar involvement’, and ‘bronchiole features’. Each parameter was graded as 0 (normal), 1 (mild), 2 (moderate) or 3 (severe). Each score was added to calculate a total score for each organ.

### Statistical analysis

A Student’s *t* test was performed to establish statistical significance between two groups. For multiple comparisons, one-way analysis of variance (ANOVA) followed by Tukey’s or Dunnett’s tests were utilized. To compare histological scores, Wilcoxon (**Figure 6F**) or Kruskal-Wallis (**Figures 6C, J**) tests were used. *p* values of <0.05 were considered significant.

## Data Availability

The original contributions presented in the study are included in the article/**Supplementary Material**. Further inquiries can be directed to the corresponding author/s.
