# Supplementary material for: Excess generation and activation of naturally arising memory-phenotype CD4+ T lymphocytes are inhibited by regulatory T cells in steady state
Source: Front Immunol. 2024 Aug 16;15:1429954. doi: 10.3389/fimmu.2024.1429954 (PMC11361994; doi:10.3389/fimmu.2024.1429954)
Supplement: Supplementary file 1 [file DataSheet1.pdf]

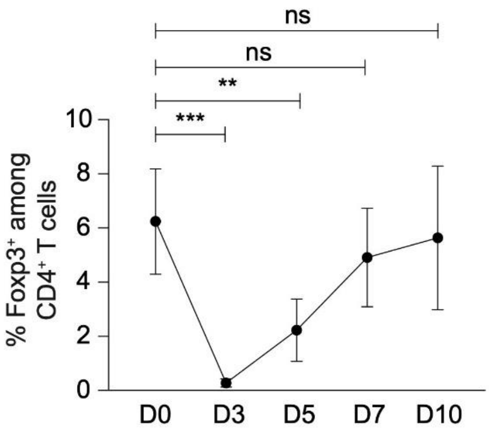

Supplementary Fig. 1. DT treatment induces transient reduction of Tregs in Foxp3-DTR mice.

Foxp3-DTR mice received DT every day and were analyzed for Foxp3^{+} cells in the blood at different time points. The graph indicates the frequency (mean ± SD) of Foxp3^{+} cells among CD4^{+} T lymphocytes at the indicated time points (n=5). ** p<0.01, *** p<0.001, ns: not significant.

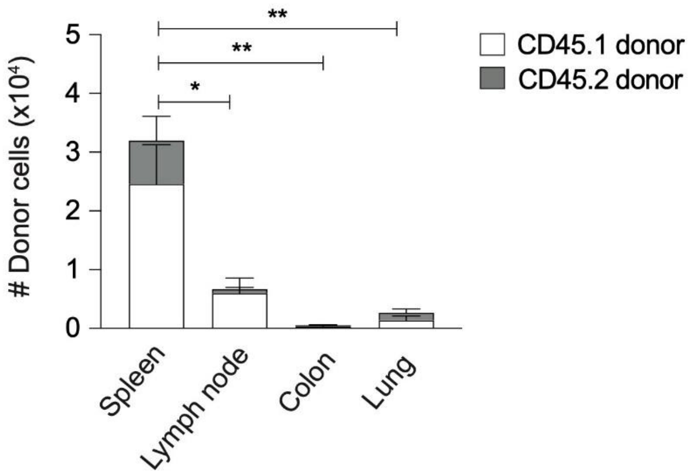

Supplementary Fig. 2. Naïve and MP donor cells preferentially accumulate in the spleen when transferred into DT-treated WT hosts.

In Fig. 1C, CD45.1 and CD45.2 donor cells were analyzed in various organs on day 2. The bar graph shows the absolute number (mean ± SD) of each donor cell population accumulating in the spleen, lymph nodes, colon, and lungs (n=3). Data are representative of two independent experiments performed. * $p<0.05$, ** $p<0.01$.

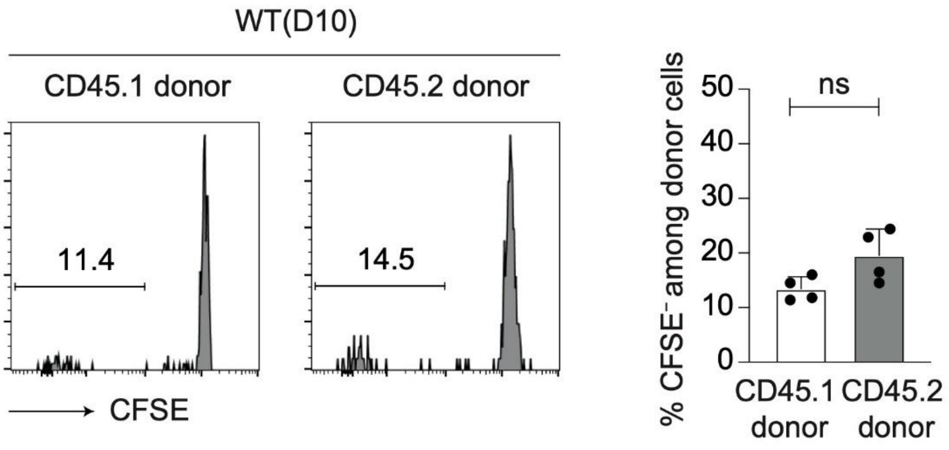

Supplementary Fig. 3. Proliferation of naïve and MP donor cells is inconspicuous in WT hosts.

In Fig. 1C, sorted and CFSE-labeled CD45.1 and CD45.2 donor cells were transferred into WT hosts that were subsequently treated with DT and analyzed on day 10. The representative histograms show CFSE dilution of donor cells whereas the bar graph indicates the frequency (mean ± SD) of $CFSE^-$ cells among each donor population (n=4). ns: not significant.

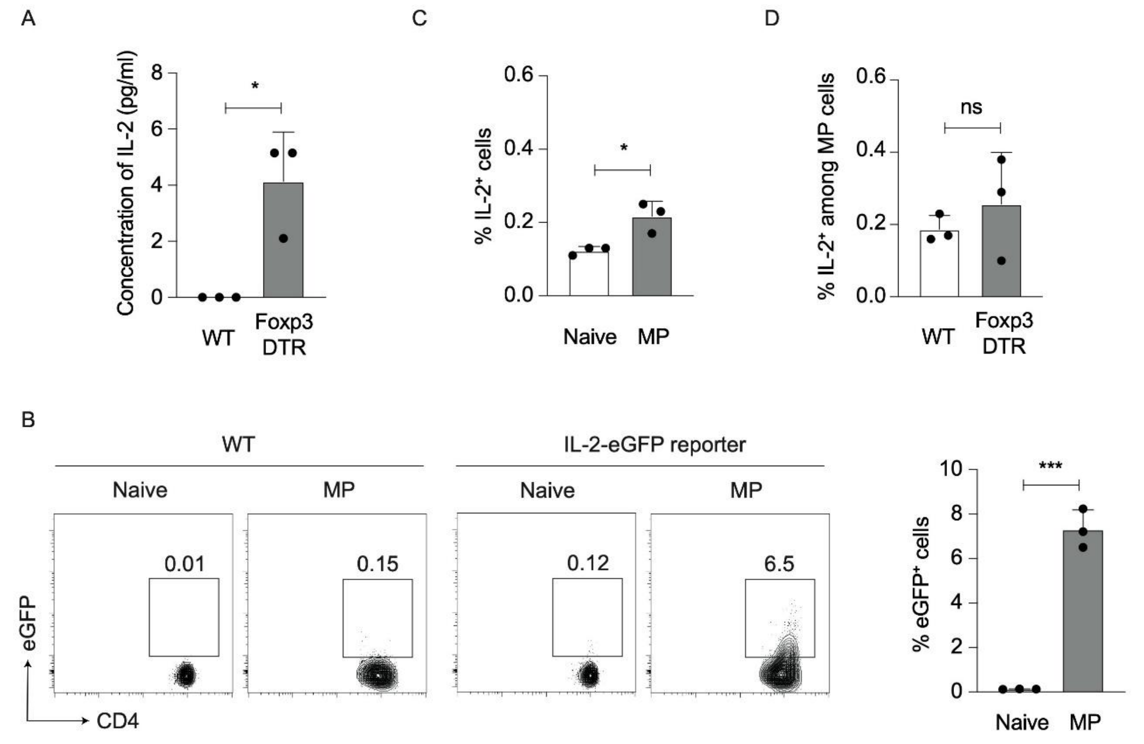

Supplementary Fig. 4. Treg depletion elevates serum IL-2 concentration without altering its expression in MP cells on a per cell basis.

(**A**) The bar graph shows the serum concentration (mean ± SD) of IL-2 in WT and Foxp3-DTR mice that were given DT for 7 days (n=3). (**B**) The representative plots display eGFP levels in MP and naïve CD4$^+$ T lymphocytes in steady-state IL-2-eGFP reporter mice whereas the bar graph indicates the frequency (mean ± SD) of eGFP$^+$ fractions among MP and naïve CD4$^+$ T cells (n=3). (**C**) The bar graph indicates the frequency (mean ± SD) of IL-2$^+$ cells among naïve and MP CD4$^+$ T cell populations in steady-state WT mice (n=3). (**D**) The graph shows the frequency (mean ± SD) of the IL-2$^+$ fractions among MP cells in DT-treated WT and Foxp3-DTR mice (n=3). Data are representative of two independent experiments. * p<0.05, *** p<0.001, ns: not significant.

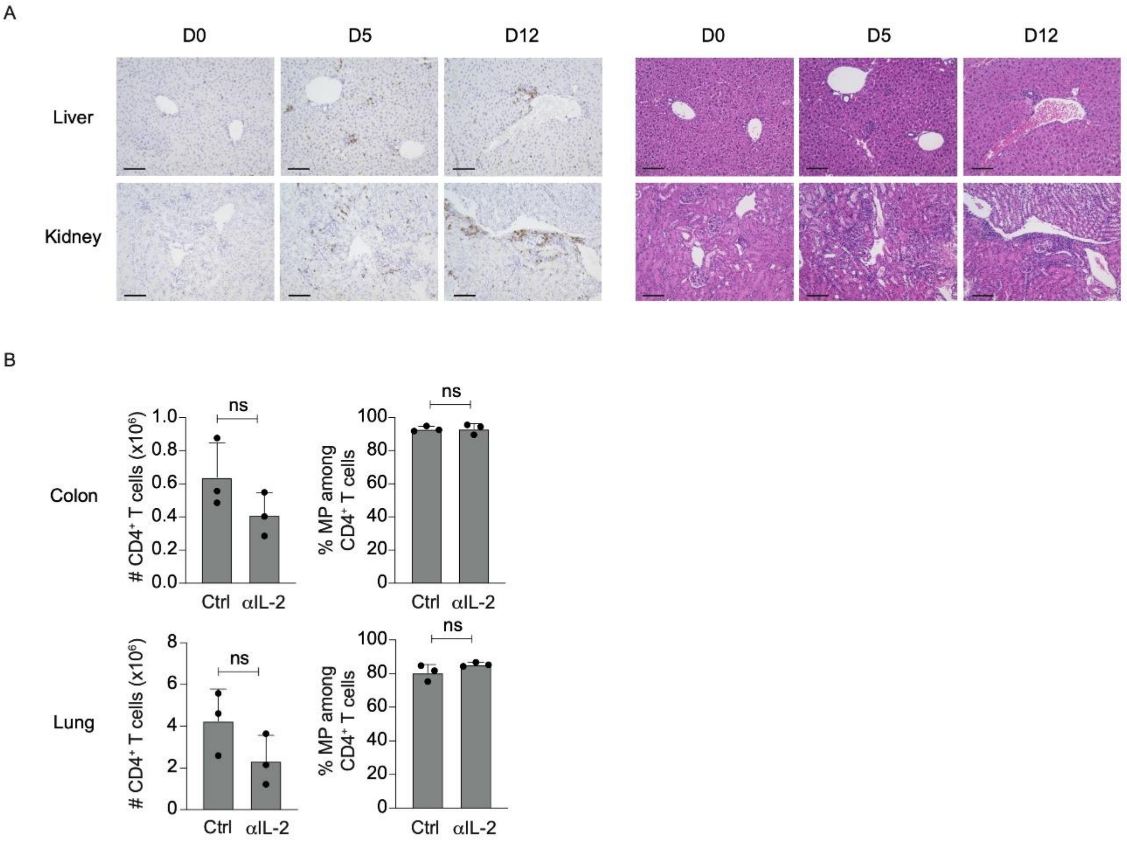

Supplementary Fig. 5. CD4^{+} T lymphocytes accumulate in multiple organs in the absence of Tregs.

(**A**) The representative images show H&E and CD4-directed immunohistochemical staining of kidneys and liver in DT-treated Foxp3-DTR mice at different time points. Data shown are representative of six mice obtained from two independent experiments performed. (**B**) The bar graphs show the absolute number (mean ± SD) of CD4^{+} T cells and their MP fractions (mean ± SD) in the indicated organs of DT-treated Foxp3-DTR mice that received anti-IL-2 mAb or control IgG for 7 days (n=3). Data are representative of two independent experiments. Scale bars: 100 µm. ns: not significant.

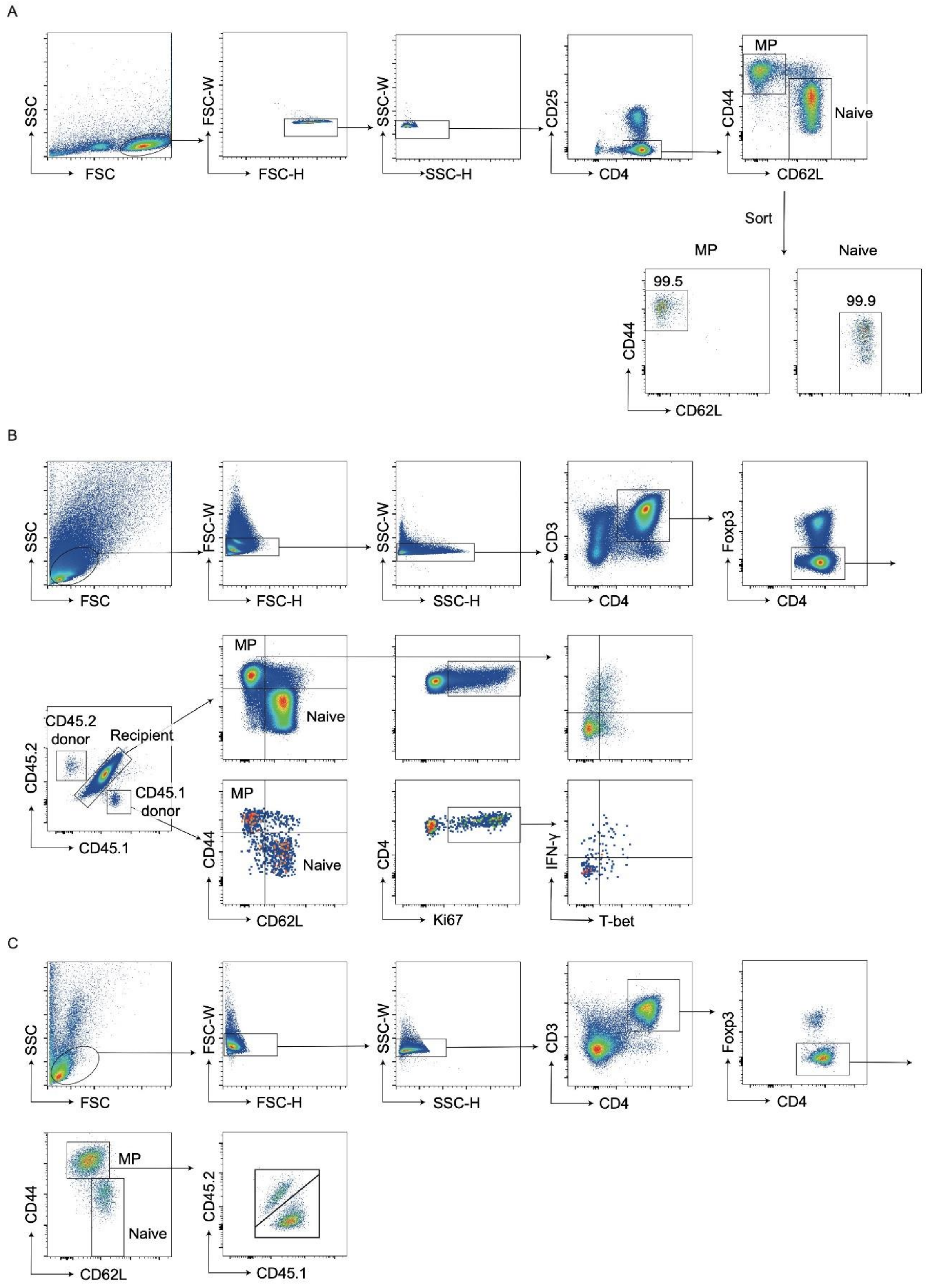

Supplementary Fig. 6. Gating strategy.

(**A**) To purify naïve and MP CD4$^+$ T cells, total singlet lymphocytes were sorted for CD4$^+$

CD25^{-} CD44^{lo} CD62^{hi} and CD4^{+} CD25^{-} CD44^{hi} CD62^{lo} subpopulations, respectively. The lower panel shows CD44 and CD62L expression in sorted cells. (**B**) To examine CD45.1 and CD45.2 donor cells in co-transfer experiments (Fig. 1C – I, 2C – D, 3, 4C – F, 5B – F), total singlet cells were gated for the CD3^{+} CD4^{+} Foxp3^{-} population. Two types of donor cells were then defined based on CD45.1 and CD45.2 expression. Gates for CD44^{hi}, CD62L^{hi}, Ki67^{+}, T-bet^{+}, and IFN-γ^{+} fractions in donor cells were determined by using recipient MP and naïve cells as a reference. (**C**) To analyze naïve and MP cells in *Rag2* KO mice in dual transfer experiments (Fig. 6G – J), total singlet cells were gated for CD3^{+} CD4^{+} Foxp3^{-} CD44^{lo} CD62L^{hi} and CD3^{+} CD4^{+} Foxp3^{-} CD44^{hi} CD62L^{lo} populations, respectively. Two types of donor cells were distinguished by CD45.1 and CD45.2 markers.
